# Circulating microRNAs miR-21-5p, miR-23a-3p and miR-26a-5p reflect clinical and molecular features of aging

**DOI:** 10.1038/s41598-025-32412-0

**Published:** 2025-12-17

**Authors:** Rossella La Grotta, Paolina Crocco, Serena Dato, Giuseppe Passarino, Giuseppina Rose

**Affiliations:** Department of Biology, Ecology and Earth Sciences, University of Calabria, 87036 Rende, CS Italy

**Keywords:** miR-21-5p, miR-23a-3p, and miR-26a-5p, Frailty, Comorbidity, Fibrosis, Biomarkers, Computational biology and bioinformatics, Diseases, Medical research

## Abstract

Circulating microRNAs (miRNAs) are emerging as key regulators of aging and age-related diseases. Among them, the so-called inflammamiRs miR-21-5p, miR-23a-3p, and miR-26a-5p have been repeatedly linked to inflammation, tissue remodelling, and metabolic dysregulation. In this cross-sectional study, we investigated their expression in older adults (65–103 years) to explore associations with frailty, comorbidity, and major clinical and functional indicators of aging. Plasma miRNA levels were measured by quantitative PCR and correlated with comprehensive clinical, functional (frailty status, Activities of Daily Living [ADL], and Cumulative Illness Rating Scale [CIRS]), and biochemical parameters. Bioinformatic pathway analyses were also performed to identify shared molecular targets. All three miRNAs showed a progressive, age-dependent increase in expression. Their associations with clinical and functional parameters remained significant after adjustment for age and sex and were confirmed in stratified analyses by sex, age, and comorbidity burden. miR-21-5p and miR-23a-3p were elevated in frail individuals, and miR-23a-3p was inversely associated with hand grip strength. miR-21-5p correlated with renal dysfunction markers, while miR-26a-5p was related to reduced ADL scores and higher comorbidity burden. Together, the three miRNAs were associated with biochemical indicators of electrolyte imbalance and systemic dysregulation, including anemia and inflammation. In silico analyses revealed convergent enrichment in the TGF-β/SMAD and RUNX1 signaling pathways, suggesting a coordinated regulatory role in inflammation-mediated fibrogenic processes. These findings identify circulating miR-21-5p, miR-23a-3p, and miR-26a-5p as potential biomarkers reflecting molecular mechanisms underlying aging and age-related decline.

**Supplementary Information:**

The online version contains supplementary material available at 10.1038/s41598-025-32412-0.

## Introduction

Aging is a complex biological process characterized by a progressive, time-dependent decline in normal physiological function, which reduces quality of life. While average life expectancy and the proportion of older individuals in the population are rising, this does not always translate to a proportional increase in disease-free lifespan or overall health ^1^. Consequently, there is growing interest in understanding the molecular mechanisms behind aging and age-related diseases to promote healthy aging by improving functional ability in later life and extending years free from illness and disability ^2^.

MicroRNAs (miRNAs) are small, non-coding, single-strand RNAs (~ 21 nucleotides) that regulate gene expression post-transcriptionally. They achieve this by binding to complementary sequences within the 3′untranslated regions (3′UTRs) of target mRNAs, promoting mRNA degradation or inhibiting translation ^3^. As key regulators of gene expression, miRNAs provide an additional layer of control in essential cellular processes like growth, differentiation, stress response, and tissue remodeling ^4^. Given their broad regulatory functions, miRNA dysregulation has been implicated in age-related functional decline and disease, including cancer, cardiovascular disease, and neurological disorders ^5–8^. Their stability in human body fluids such as serum and plasma coupled with evidence of their changes with age-related pathologies suggests their potential as non-invasive biomarkers of aging.

Within this context, significant attention has been given to inflamma-miRNAs ^9^, a class involved in regulating the inflammatory response. The aging process is well-known to be accompanied by a low-grade systemic chronic inflammatory state, termed "inflamm-aging" ^10^. This state arises from prolonged immune pathway activation, elevated pro-inflammatory cytokine production, and disrupted immunological homeostasis ^11^. Such chronic low-grade inflammation often precedes most disease conditions, profoundly affecting basic biological processes. It impairs essential cellular functions like the response to oxidative stress, mitochondrial function, and DNA repair capacity, while also promoting cellular senescence and tissue fibrosis ^12–14^. These alterations trigger a cascade of deleterious effects, increasing the risk and promoting the onset of numerous age-related diseases, including type 2 diabetes, chronic kidney disease, cardiovascular disease ^15,16^.

Building on this evidence, the present study focused on three specific miRNAs: miR-21-5p (17q23.1), miR-23a-3p (19p13.12, part of the miR-23a ~ 27a ~ 24–2 cluster), and miR-26a-5p (3p23). These highly conserved miRNAs have been extensively researched due to their involvement in various pathological conditions. They were selected primarily due to their well-established roles in both inflammatory responses ^17–19^ and crucial aging-related mechanisms. Specifically, they play a role in key regulatory pathways, including apoptosis ^20–22^, cellular senescence ^23–25^, mitochondrial dysfunction ^26–28^, immune dysregulation ^29–31^ and functional decline ^32^. Given these multiple implications, these miRNAs have garnered significant interest in the context of aging research. Therefore, this study aimed to explore the plasma expression profiles of these three specific miRNAs in a cohort of older individuals (aged 65–103 years), assessing their potential association with clinical, functional, and laboratory parameters to enhance our understanding of their role in overall health.

## Materials and methods

### Participants

A total of 208 participants, aged 65–103 years (mean age: 83.8 ± 7.33 years), were included in the study. Of these, 149 were female (range: 65–103 years, mean age: 84.3 ± 7.29 years) and 59 were male (range: 65–97 years, mean age: 82.6 ± 7.32 years).

Samples were collected from elderly nursing homes in Calabria, southern Italy, as part of a study designed to assess the quality of aging in the region. A multidimensional geriatric evaluation was conducted to assess the social, cognitive, physical and functional abilities of all participants. This was achieved by collecting data through the implementation of a structured questionnaire administered by a trained operator. A peripheral blood sample was obtained from each participant for laboratory examinations, including traditional biomarkers and miRNAs.

The study received ethical approval from the local Ethics Committee "Comitato Etico Regione Calabria-Sezione Area Nord" on 31 October 2017 (code n. 25/2017). Written informed consent was obtained from each subject before enrollment in the study. When appropriate, a relative or legal representative signed the consent on behalf of participants with limited cognitive capacity.

### Anthropometric, laboratory, and clinical assessments

Participant measurements were conducted by trained nurses. Waist and hip circumferences were taken to calculate the waist-to-hip ratio (WHR). Height and weight, measured without shoes and in light clothing, were used to determine body mass index (BMI) using the formula: weight (kg) / height (m^2^). Blood pressure (BP), including systolic (SBP) and diastolic (DBP) readings, was measured three times on the right arm after at least five minutes of rest, and the average of these measurements was recorded. Routine hematological and biochemical markers, detailed in Table 1, were analyzed at the Italian National Research Center on Aging (Cosenza) following standardized protocols. The glomerular filtration rate (GFR) was estimated using the creatinine-based Berlin Initiative Study 1 (BIS1) equation, which is specifically designed for older adults: eGFR-BIS1 = 3736 × creatinine ^−0.87^ × age^−0.95^ × 0.82 (if female). The GFR was then analysed both continuously and dichotomously, categorizing subjects as having either moderate-to-severe renal dysfunction (eGFR ≤ 45 ml/min/1.73 m^2^) or not. Hypertension was defined as SBP/DBP ≥ 140/90 mmHg or the use of antihypertensive medication. Diabetes was defined as fasting plasma glucose > 125 mg/dL or the use of antidiabetic therapy. The presence of cardiovascular disease (CVD) was determined through a comprehensive review of medical history, clinical symptoms, imaging results, and physical or laboratory examinations, as evaluated by a board-certified cardiologist following international clinical guidelines.

**Table 1:** Baseline characteristics of the older cohort analysed (N = 208).

Category	Characteristic	Value
Demographic and anthropometric	Median Age (years)	83.85 (7.33)
	Females (%)	71.6
	BMI (kg/m^2^)	23.81 [20.86, 28.57]
Clinical	Blood Glucose (mg/dL)	88.00 [80, 105.30]
	HbA1c (%)	5.70 [5.20, 6.40]
	Triglycerides (mg/dL)	107 [83, 134.00]
	Total Cholesterol (mg/dL)	154.03 (40.66)
	HDL-Cholesterol (mg/dL)	47.51 (13.62)
	LDL-Cholesterol (mg/dL)	84.67 (33.61)
	eGFR	48.44 (14.01)
	Systolic BP (mmHg)	130 [115, 140]
	Diastolic BP (mmHg)	70 [70, 80]
Hematological parameters	RBC (× 10^12^/L)	4.19 (0.63)
	WBC (× 10⁹/L)	6.60 [5.45, 7.80]
	Lymphocytes (%)	24.78 (10.05)
	Neutrophils (%)	60.2 [53.20, 66.6]
	Eosinophils (%)	2.20 [1.10, 3.60]
	Basophils (%)	0.80 [0.50, 1.10]
	Monocytes (%)	11.07 (5.5)
	Platelets (× 10⁹/L)	228 [186.5, 287.5]
	Hemoglobin (g/dL)	12.20 [10.8, 13.3]
	Hematocrit (%)	36.40 [33.42, 40.3]
Electrolytes and minerals	Sodium (mEq/L)	141.00 [139, 142]
	Potassium (mEq/L)	4.5 (0.58)
	Chloride (mEq/L)	105 [102, 107]
	Calcium (mg/dL)	9.045 (0.61)
	Magnesium (mg/dL)	1.90 [1.70, 2.10]
	Phosphorus (mg/dL)	3.50 [3.10, 3.90]
Biochemical and inflammatory markers	Albumin (g/dL)	52.76 (7.27)
	Total Protein (g/dL)	6.50 [6.20, 6.90]
	BUN (mg/dL)	45 [35, 59.5]
	Uric Acid (mg/dL)	4.40 [3.70, 5.40]
	Creatinine (mg/dL)	1.00 [0.87, 1.24]
	Bilirubin (mg/dL)	0.60 [0.44, 0.85]
	Ferritin (ng/mL)	121 [54.5, 215.3]
	CRP (mg/L)	4.80 [1.90, 19.75]
Morbidity	CIRS score	9.00 [6.00, 16.00]
	MMSE score	16.36 (6.47)
	Hand Grip Strength (kg)	12.15 [8.53, 18.00]
	ADL dependence (%)	72.5
	Frailty (%)	63.5
	Diabetes (%)	26.0
	Hypertension (%)	71.6
	Cardiovascular Disease (%)	46.6

Data are presented as mean (SD), median [IQR], or % as appropriate. *Abbreviations:* BMI = Body Mass Index, HbA1C = Glycosylated Hemoglobin, LDL = Low-density lipoprotein, HDL = High-density lipoprotein, eGFR = Estimated Glomerular Filtration Rate, RBC = Red Blood Cells, WBC = White Blood Cells, BUN = Blood,Urea Nitrogen, CRP = C-Reactive Protein, CIRS = Cumulative Illness Rating Scale, MMSE = Mini Mental State Examination; ADL = Activities of Daily Living.

### Geriatric evaluation

Physical performance was assessed by hand grip (HG) strength, a measure of muscle strength obtained by a handheld dynamometer (SMEDLEY’s dynamometer TTM). Subjects were seated with the elbow flexed at 90^∘^ and the wrist maintained in a neutral position. Three measurements were performed with both hands, with a 30-s rest interval established between consecutive measurements. The highest value recorded was used for analysis. All measurements were conducted during the morning hours (between 9:00 and 11:00 AM) to minimize diurnal variations in muscle strength. Functional activity was measured using the Activities of Daily Living (ADL) score ^33^. The number of activities (bathing, dressing, using the toilet, transferring from bed to chair, and feeding) in which the participant is dependent or independent at the time of the visit is used to calculate the score. For the analysis, ADL scores were dichotomized as one if the subject was not independent in all five items and zero otherwise. Cognitive function was assessed using the Mini-Mental State Examination (MMSE), which evaluates language, visuospatial abilities, attention, orientation, and episodic memory ^34^. MMSE scores range from 0 to 30, with adjustments made for age and educational level to ensure normalization ^35^.

Frailty was determined using a multidimensional cluster-based approach based on the methodology described by Montesanto et al. ^36^. This method involves applying Hierarchical Cluster Analysis (HCA) to standardized measures from three essential geriatric domains, ensuring alignment with widely accepted frailty concepts: (i) Cognitive Function, assessed using the Mini Mental State Examination (MMSE); (ii) Functional Capacity, evaluated through the Activities of Daily Living (ADL); and (iii) Physical Performance, measured by handgrip strength.

The HCA was applied to the combined data from these three domains, allowing the frailty groups to emerge from the population structure without requiring the imposition of arbitrary, pre-defined clinical cut-offs. Based on the characteristics and size of our cohort, the analysis yielded two distinct and clearly separable clusters: non-frail subjects (characterized by better overall performance across the domains) and frail subjects (displaying poorer average scores). This cluster-based approach has been previously validated in survival analyses across independent European populations, supporting its robustness and reproducibility ^36,37^.

Multimorbidity was assessed using the modified Cumulative Illness Rating Scale (CIRS), which evaluates the burden of chronic conditions across 14 physiological systems (cardiac, vascular, respiratory, gastrointestinal, hepatic, renal, genitourinary, musculoskeletal, dermatologic, neurologic, endocrine, metabolic, breast, and psychiatric) ^38,39^. Each system received a score from 0 (no issue) to 4 (severe impairment), resulting in total scores ranging from 0 to 56, with higher scores indicating greater overall morbidity.

### Total RNA extraction and cDNA synthesis

Venous blood samples were collected from all participants in the morning, after an overnight fast by venipuncture into EDTA-containing tubes. Plasma was separated by centrifugation at 1800 × g for 10 min at room temperature, then transferred to RNase-free tubes and further centrifuged at 1200 × g for 20 min at 10 °C to remove any cellular contaminants. To prevent freeze–thaw cycles, plasma was divided into aliquots and stored at − 80 °C until analysis.

Total RNA was extracted from the plasma samples by using the mirVana™ PARIS™ RNA and Native Protein Purification Kit (Cat. No. AM1556, Thermo Fisher Scientific) according to the manufacturer’s instructions. Briefly, 200 μl of plasma was mixed with an equal volume of 2 × denaturing solution, followed by the addition of 25 fmol of synthetic cel-miR-39-3p (478293_mir, Thermo Fisher Scientific) as the spike-in control. After the organic extraction with acid–phenol and chloroform, 1.25 volumes of room temperature 100% ethanol were added to the aqueous phase. Three washes were performed before eluting the RNA with 100 μl of preheated elution solution at 95 °C.

The cDNA template was prepared using the TaqMan™ Advanced miRNA cDNA Synthesis Kit (Cat No. A25576, Applied Biosystems), and 2 μl of eluted RNA was used for two-step PCR amplification according to the user’s manual. Specifically, the following steps were performed: Poly(a) tailing addition, 5’-end adaptor-ligation, reverse transcription and miRNA universal pre-amplification using primers provided in the kit. The resulting cDNA product was stored at − 20 °C until final detection by qRT-PCR.

### miRNA quantification

Quantitative real-time PCR (qPCR) was carried out to quantify miRNA by loading 5 μl of 1:10 diluted pre amplified cDNA, 10 μl TaqMan™ Fast Advanced Master Mix, no UNG (A44360, Applied Biosystems), and 1 μl of Taqman advanced assay specific for each of the examined miRNAs was as follows: hsa-mir-21-5p (ID: 477975); hsa-mir-23a-3p (ID: 478532); hsa-mir-26a-5p (ID: 477995). We performed qPCR on QuantStudio3™ Real-Time PCR System (Applied Biosystems, Milan, Italy) with the following settings: 95 °C for 20 s, followed by 40 cycles of 95 °C for 1 s and 60 °C for 20 s. For each miRNA assay, a no-template control containing nuclease-free water instead of a miRNA probe was used. All samples were run in triplicate considering a threshold cycle (Ct) < 35.

Hsa-miR-484 (ID: 478308) was used as endogenous control for qPCR data normalization, as previous studies have demonstrated its stability in plasma/serum ^40^. In our cohort, Ct values of miR-484 did not differ significantly across age, sex, or clinical categories, confirming its suitability as a reference.

Relative plasma levels for each miRNA were calculated by normalizing expression levels to the reference miRNA using the comparative threshold (Ct) method 2^−ΔCt ^^41^.

To ensure a better fit for a normal distribution, the miRNAs expression levels were log-transformed.

### miRNA pathway analysis

To identify the pathways and targets associated with the microRNAs that constituted the subject of the study, the online platform DIANA-miRPath (v4.0, http://www.microrna.gr/miRPathv4) was employed. Using computationally predicted or experimentally validated interaction data, this tool enables analysis of the combined effects of miRNAs ^42^. Interactions supported by experiments were obtained from the DIANA-TarBase v8 database, and pathway enrichment analysis was performed using the Reactome database. The miRNAs were subjected to miRNA-centric analysis using the “Genes union”, “Genes intersection” and “Pathways union” merging methods, with statistical significance set at *p* < 0.05 and false discovery rate (FDR) correction applied.

### Statistical analysis

Shapiro–Wilk test was used to evaluate the distribution of variables. Categorical variables are reported as percentages, continuous variables as means (standard deviation) for normal distributed variables, and median (interquartile range) for non-normal distributed variables. The Pearson’s test was used to determine the correlation among the plasma levels of three analysed miRNAs, as well as correlation with age. Linear regression was used to test the strength of association between miRNAs levels and age while controlling for sex. Multivariate regression analyses adjusted for age and sex, were employed to explore the relationship between the expression levels of the investigated miRNAs and the selected variables. Non-normally distributed variables were log-transformed prior to inclusion in regression analysis. To further explore subgroup effects, participants were stratified by sex, age, and comorbidity status. For stratified analyses, age was dichotomized into two groups (below and above the median), while comorbidity was categorized as low or high based on the median CIRS score. For each stratification, ANCOVA models were applied to assess differences in circulating miRNA levels across subgroups, adjusting for relevant covariates (age, sex, or both, as appropriate). The assumption of homogeneity of regression slopes was tested for all models. In addition, to assess the linearity of the associations between miRNA levels and age, participants were also divided into age tertiles, and differences among tertiles were tested using ANCOVA adjusted for sex.

All reported *p*-values were two-sided, and *p*-values lower than 0.05 were considered to indicate statistical significance. All statistical data were analyzed with R version 4.3.3 (2024-02-29).

## Results

Table 1 summarizes the anthropometric, biochemical, and clinical characteristics of the 208 participants. As the Table shows, the gender distribution was imbalanced, with males making up 28.4% of the cohort and females making up the remaining 71.6%.

### miRNAs in relation to physical and cognitive functioning and multimorbidity

**Fig. 1:**
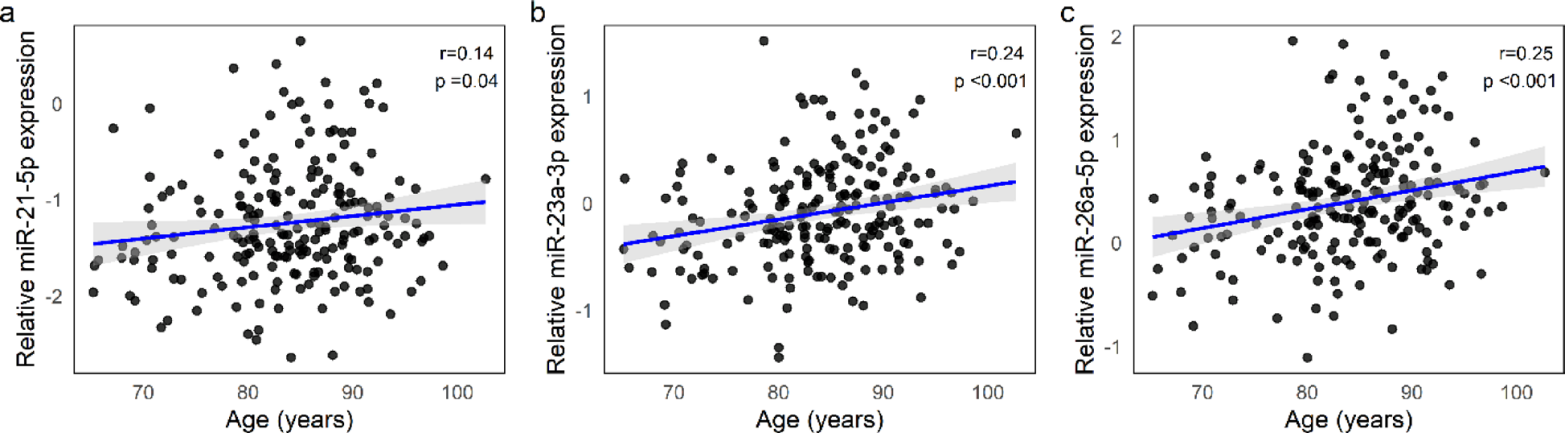
Correlation plots between age and the relative expression of (**a**) miR-21-5p, (**b**) miR-23a-3p, and (**c**) miR-26a-5p. Black dots represent individual data points; blue lines indicate linear regressions with 95% confidence intervals (grey shading). Pearson’s r and *p*-values are shown in each panel. Relative miRNA expression is calculated as (2^ − ΔCq^).

To evaluate possible age-related modifications of the expression levels of the three miRNAs tested, we first performed a correlation analysis. Significant positive correlations with age were found for miR-21-5p (r = 0.14, *p* = 0.04), miR-23a-3p (r = 0.24, *p* < 0.001), and miR-26a-5p (r = 0.25, *p* =  < 0.001) (see Fig. 1a–c). These results were confirmed by regression analyses controlling for sex (*p* = 0.04 for miR-21-5p, *p* < 0.001 for miR-23a-3p and miR-26a-5p). To assess whether the associations with age were driven by specific subgroups, participants were divided into age tertiles. Both miR-23a-3p and miR-26a-5p showed significantly higher expression in the oldest age tertile compared to the youngest (*p* < 0.001 for both), whereas miR-21-5p did not differ significantly across tertiles. These results reflect a gradual increase across the age range.

**Fig. 2:**
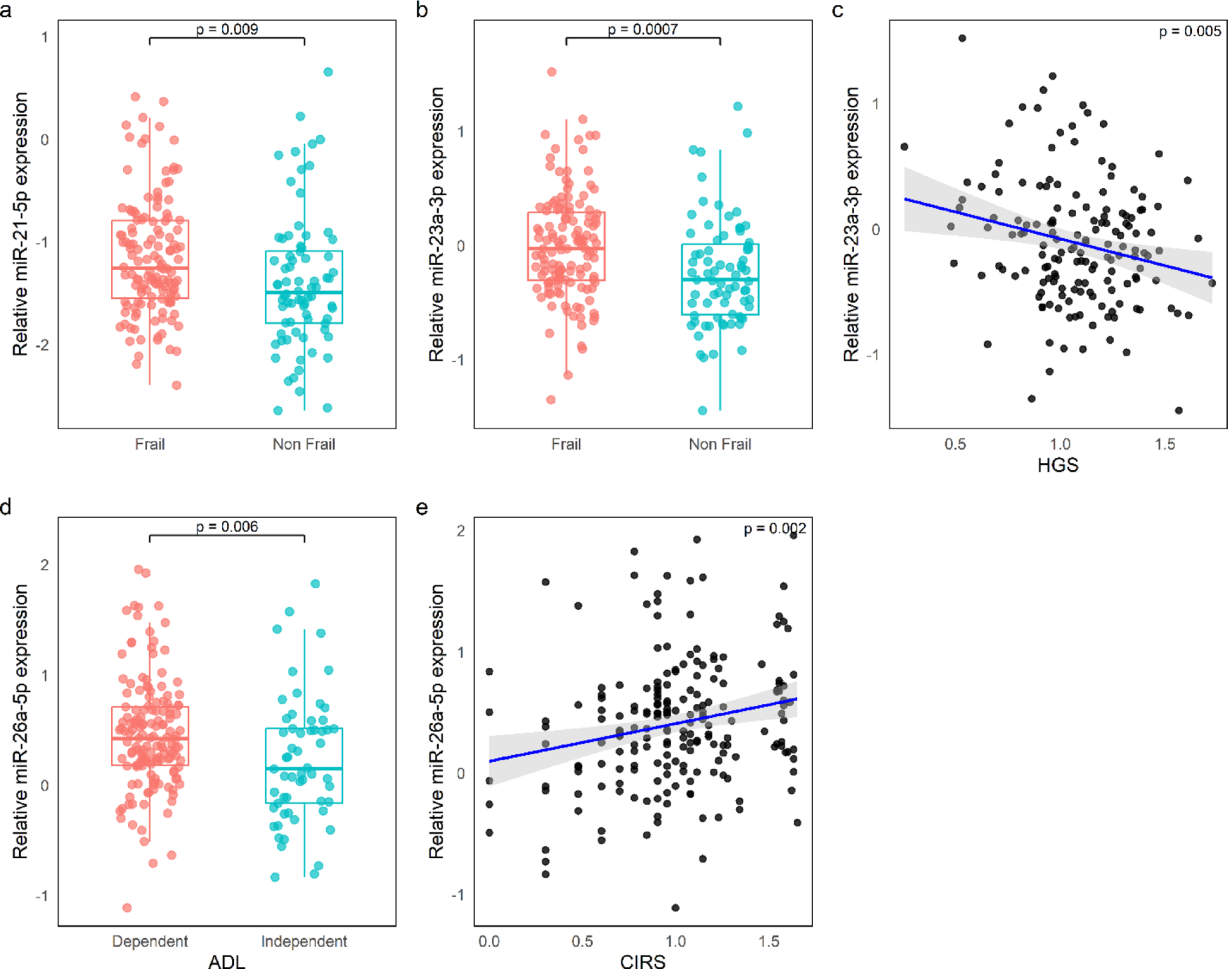
Relative expression of selected miRNAs in relation to functional parameters. (**a**–**b**) Expression of miR-21-5p and miR-23a-3p in Frail (red) vs. non-Frail (blue) individuals. (**c**) Negative correlation between miR-23a-3p expression and Hand Grip Strength (HGS) (log-transformed); (**d**) Expression of miR-26a-5p in individuals dependent vs. independent in Activities of Daily Living (ADL); (**e**) Positive correlation between miR-26a-5p expression and Cumulative Illness Rating Scale (CIRS, log-transformed). Relative miRNA expression is calculated as log(2^ − ΔCq^).

In addition, we found that the three miRNAs were significantly and positively correlated with one. another (*p* < 0.001 for all combinations), suggesting potential co-expression or shared regulatory mechanisms. To investigate their clinical relevance, we first performed unadjusted linear and logistic regression analyses to assess crude associations between miRNA expression levels and key geriatric parameters, including physical and cognitive functioning, functional status, and multimorbidity. Statistically significant results from these exploratory analyses (*p* < 0.05) are presented in Fig. 2, which includes only miRNAs showing relevant associations. In particular, miR-21-5p and miR-23a-3p were upregulated in frail individuals; additionally, higher miR-23a-3p expression was associated with reduced HG strength. miR-26a-5p expression, on the other hand, was associated with impaired autonomy in basic activities of daily living and with a higher multimorbidity burden.

To account for potential confounding factors, we then performed multivariable regression analyses adjusted for sex and age. The results, reported in Table 2, support the findings of the unadjusted models, indicating that the observed associations were robust. Specifically, miR-21-5p and miR-23a-3p remained significantly upregulated in frail compared to non-frail individuals (Fig. 2a and b), with adjusted odds ratios of 1.77 (95% CI: 1.02–3.1; *p* = 0.04) and 2.45 (95% CI: 1.16–5.14; *p* = 0.018), respectively. Furthermore, higher miR-23a-3p expression was significantly associated with reduced HG strength (β = − 0.10, *p* = 0.005; Fig. 2c), suggesting a potential role in physical decline. Similarly, miR-26a-5p expression was associated with functional impairment and multimorbidity. As shown in Fig. 2d and e, higher levels were observed in individuals with reduced ability to perform basic ADLs and with increased CIRS scores. These associations remained significant after adjustment for covariates (ADL: OR = 1.99, 95% CI = 1.03–3.84, *p* = 0.041; CIRS: β = 0.12, *p* = 0.010).

**Table 2:** Significant associations between multidimensional geriatric assessment parameters and plasma miRNA levels.

miRNA	Frailty		HGS^a^		ADL		CIRS^a^	
	*OR (CI)*	*p*	*Β (SE)*	*p*	*OR (CI)*	*p*	*β (SE)*	*p*
miR-21-5p	1.77 (1.02–3.1)	0.040						
miR-23a-3p	2.45 (1.16–5.14)	0.018	− 0.10 (0.04)	0.005				
miR-26a-5p					1.99 (1.03–3.84)	0.041	0.12 (0.05)	0.010

(a) log-transformed parameters.

Results are derived from logistic and linear regression models, adjusted for sex and age. For binary outcomes, odds ratios (ORs) with 95% confidence intervals (CIs) and *p*-values are reported. For continuous outcomes, beta coefficients (β) and *p*-values are presented. Only associations that remained statistically significant (*p* < 0.05) after adjustment are shown. Empty cells indicate non-significant associations.

*Abbreviations*: HGS = Hand Grip Strength, ADL = Activities of Daily Living, CIRS = Cumulative Illness Rating Scale.

### Correlation analysis between miRNAs expression levels and biochemical and clinical variables

Building on these findings, we next examined the associations between the three miRNAs and circulating biochemical markers to gain further insights into their potential mechanistic roles. Results of linear and logistic regression analyses, adjusted for age and sex, are presented in Table 3 and detail the strength and significance of the observed associations. Only results that remained significant after adjustment for covariates are reported.

**Table 3:** Associations between circulating miRNAs and biochemical and hematological parameters.

miRNA	BUN^a^	sCr^a^	eGFR	TP^a^	K	LYM	RBC	HCT^a^	Hb^a^
	β (SE)	β (SE)	β (SE)	β (SE)	β (SE)	β (SE)	β (SE)	β (SE)	β (SE)
miR-21-5p	0.11 (0.05) *p*= 0.027	0.08 (0.04) *p* = 0.036	− 3.41 (1.46) *p* = 0.021	− 0.03 (0.01) *p*= 0.016		3.02 (1.11) *p*= 0.007	− 0.16(0.07) *p*= 0.020	− 0.04 (0.02) *p*= 0.019	− 0.04 (0.02) *p* = 0.032
miR-23a-3p				− 0.04 (0.01) *p* = 0.012	0.2 (0.09) *p* = 0.02		− 0.18(0.09) *p* = 0.048	− 0.06 (0.02) *p* = 0.016	− 0.06 (0.02) *p* = 0.026
miR-26a-5p	0.15 (0.06) *p* = 0.009			− 0.04 (0.01) *p* = 0.0016	0.21 (0.08) *p* = 0.008			− 0.06 (0.02) *p* = 0.008	− 0.06 (0.02) *p* = 0.007

(^a^) log-transformed parameters.

Results are derived from linear regression models, adjusted for sex and age. Beta coefficients (β), their standard errors (SE) in parentheses, and *p*-values are.

presented. Only associations that remained statistically significant (*p* < 0.05) after adjustment are shown. Empty cells indicate non-significant associations.

*Abbreviations:* BUN = Blood Urea Nitrogen, sCr = Serum Creatinine, eGFR = Estimated Glomerular Filtration Rate, TP = Total Protein, K = Potassium, LYM = Lymphocytes, RBC = Red Blood Cells, HCT = Hematocrit, Hb = Hemoglobin.

**Fig. 3:**
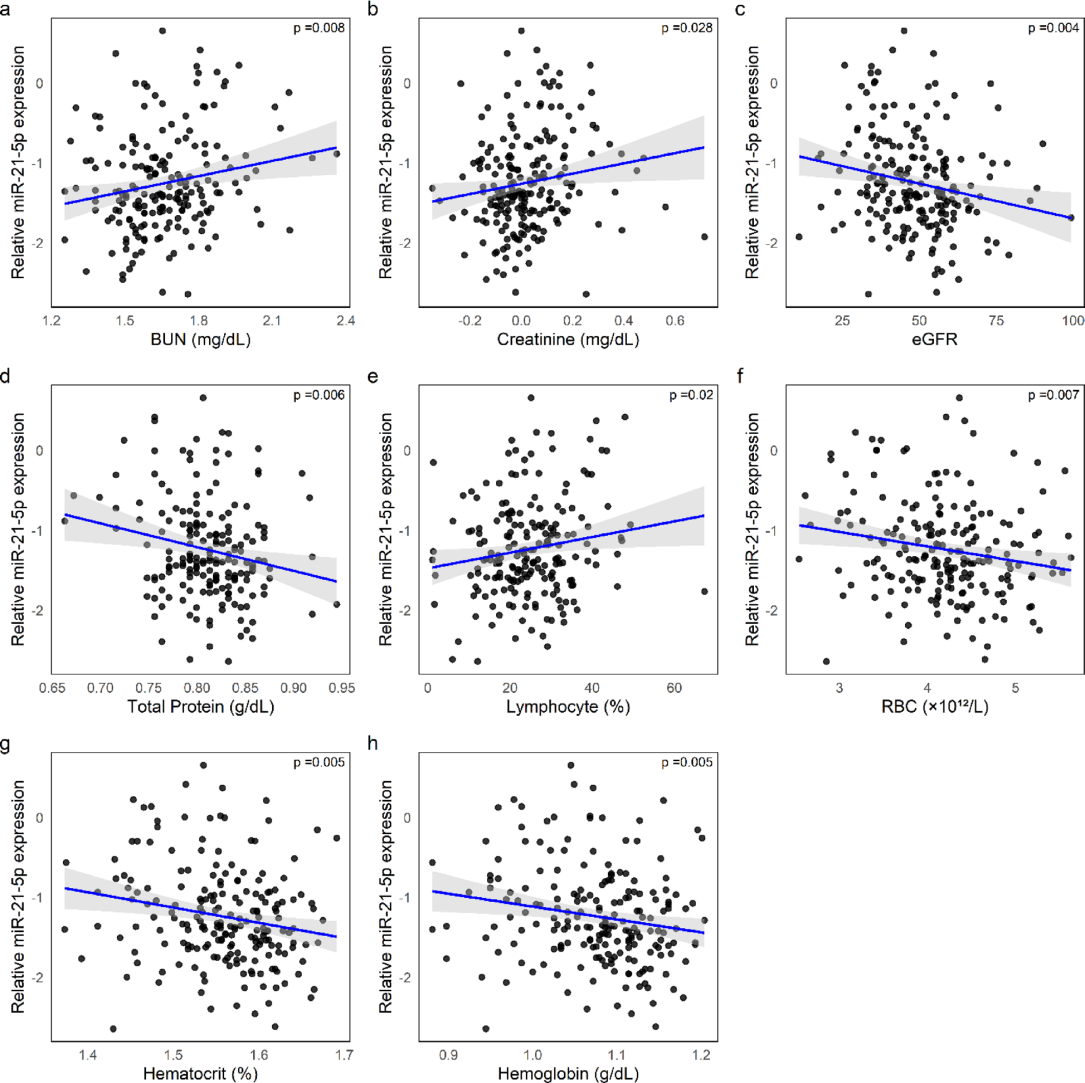
Associations between miR-21-5p expression and clinical parameters. (**a**–**h**) Scatter plots showing the relative expression of miR‑21‑5p [log(2^ − ΔCq^)] in relation to various biochemical and hematological parameters: Blood Urea Nitrogen (BUN, log-transformed) (**a**) and creatinine (log-transformed) (**b**) show positive correlations; Estimated glomerular filtration rate (eGFR) (**c**), Total Protein (log-transformed) (**d**), red blood cell count (RBC) (**f**), hematocrit (log-transformed) (**g**), and hemoglobin (log-transformed) (**h**) show negative correlations; while lymphocyte percentage (**e**) is positively correlated. Blue lines indicate linear regressions with 95% confidence intervals (grey shading).

As shown in Fig. 3a–h, higher expression levels of miR-21-5p were significantly associated with increased blood urea nitrogen (BUN) and serum creatinine, and with decreased estimated glomerular filtration rate (eGFR), markers indicative of impaired renal function. Logistic regression using dichotomized eGFR values (cut-off < 45 ml/min/1.73 m^2^, reflecting moderate-to-severe renal impairment) yielded an odds ratio of 2.04 (95% CI: 1.23–3.38, *p* = 0.006), further supporting the link between miR-21-5p and renal dysfunction. In addition, miR-21-5p expression was significantly associated with lower total protein levels and hematological and inflammatory markers, including a higher lymphocyte percentage and lower red blood cell (RBC) count, hematocrit (HCT) and hemoglobin (Hb).

**Fig. 4:**
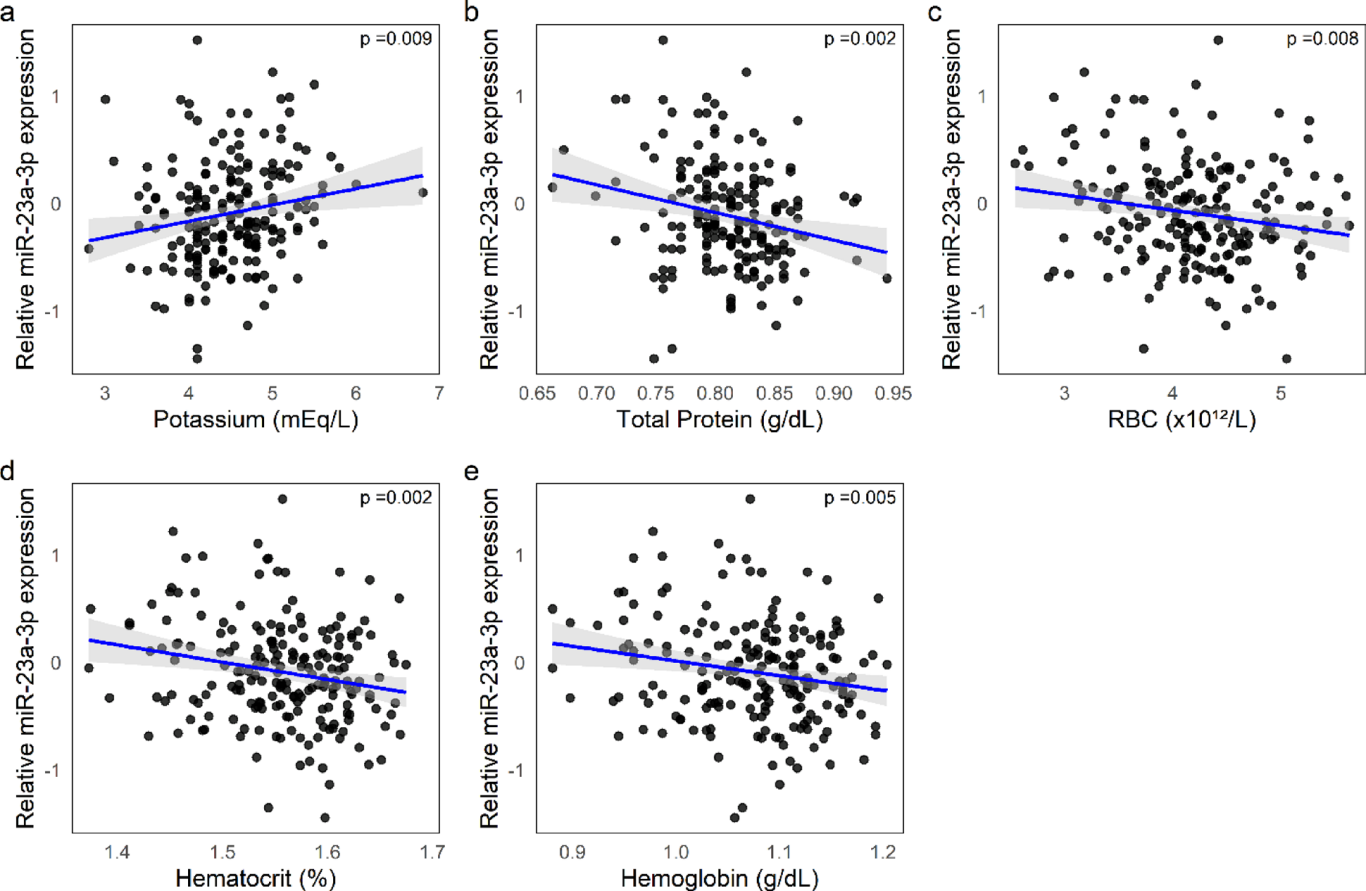
Associations between miR-23a-3p expression and clinical parameters. (**a**–**e**) Scatter plots showing the relative expression of miR‑23a‑3p [log(2^ − ΔCq^)] in relation to: Potassium (**a**), positive correlation; Total Protein (log-transformed) (**b**), Red blood cell count (RBC) (**c**), Hematocrit (log-transformed) (**d**), and Hemoglobin (log-transformed) (**e**), all showing negative correlations. Blue lines represent linear regressions with 95% confidence intervals (grey shading).

**Fig. 5:**
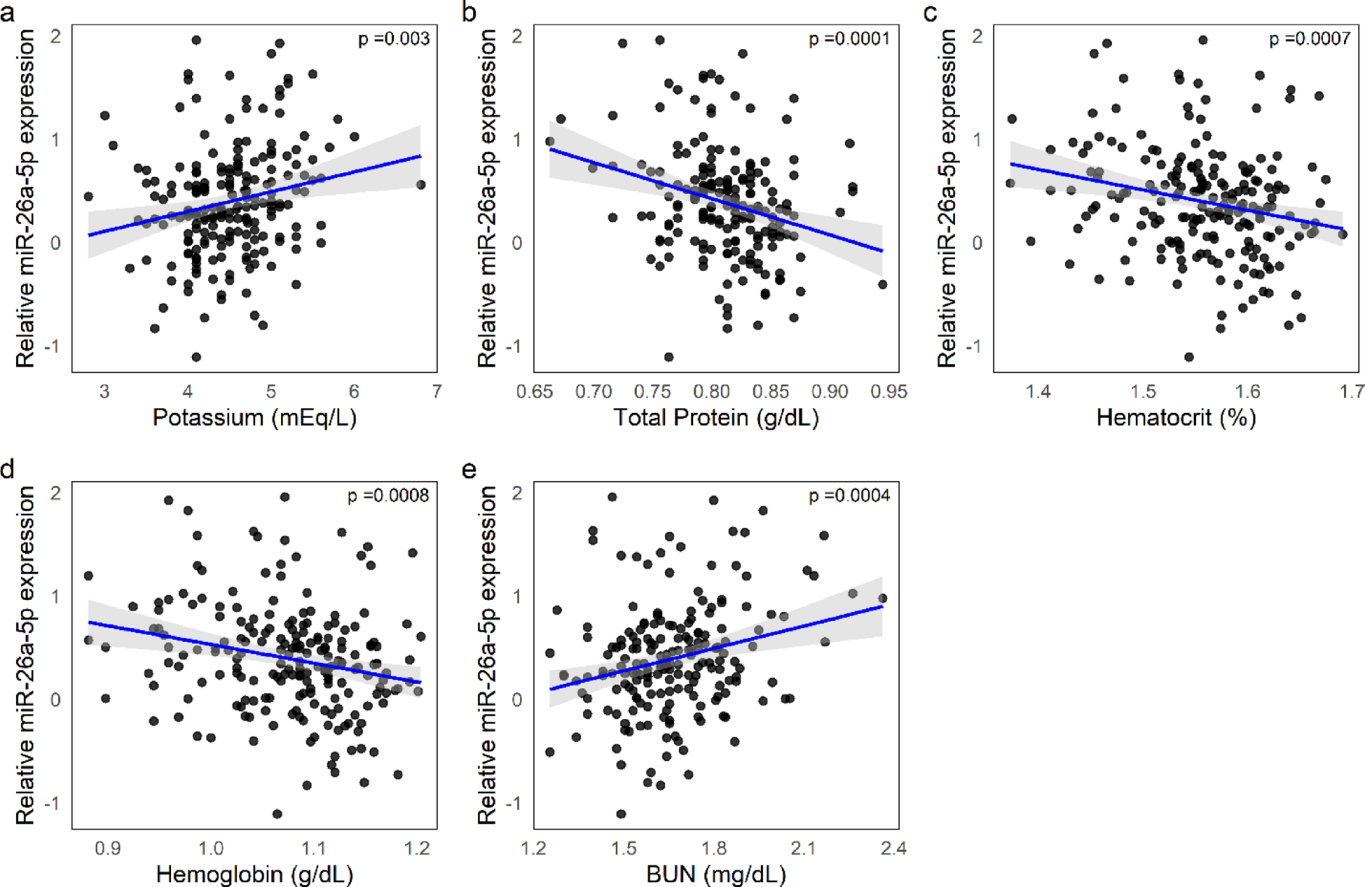
Associations between miR‑26a‑5p expression and clinical parameters. (**a**–**e**) Scatter plots showing the relative expression of miR-26a-5p [log(2^ − ΔCq^)] in relation to: Creatinine (**a**) and Cumulative Illness Rating Scale (CIRS) score (**b**), both showing positive correlations; Total Protein (log-transformed) (**c**), Red blood cell (RBC) count (**d**), and Hemoglobin (**e**), all showing negative correlations. Each plot includes a linear regression line (blue) with 95% confidence intervals (grey shading).

MiR-23a-3p showed a positive association with serum potassium and a negative association with total protein, as well as with hematological indices such as RBC, HCT, and Hb (see Fig. 4a–e). A similar pattern was observed for miR-26a-5p, which also showed a positive correlation with BUN (see Fig. 5a–e).

To evaluate the robustness of the observed associations and potential subgroup-specific effects, additional ANCOVA analyses were performed after stratifying participants by sex (adjusted for age), age group (below vs above median age, adjusted for sex), and comorbidity level (low vs high CIRS score, adjusted for age and sex). As reported in Supplementary Tables S1, S2, S3, the direction and strength of the associations remained consistent across all subgroups, confirming the stability of the observed miRNA–phenotype relationships. In females, associations mirrored the overall cohort; in males, trends were similar but weaker, likely due to limited power. A significant interaction between sex and ADL was observed for miR-26a-5p, suggesting that the link between miR-26a-5p expression and functional dependence may differ between men and women. No other significant interaction effects were detected.

### Pathway enrichment analysis

To comprehensively understand the biological functions influenced by miR-21-5p, miR-23a-3p, and miR-26a-5p, we performed a pathway enrichment analysis using the Reactome database in DIANA-miRPath v4.0. The “Gene Union” analysis, which examined genes targeted by at least one of three miRNAs, revealed a wide range of significantly enriched pathways, highlighting the substantial impact of this miRNA set on fundamental cellular machinery. Notably, gene expression, membrane trafficking, protein modification and metabolism, were among the most enriched terms. See Supplementary Fig. S1 for a complete list.

A “gene intersection” analysis was then performed to identify pathways commonly enriched by the target genes of miR-21-5p, miR-23a-3p, and miR-26a-5p. This analysis holds significant importance as our study revealed a positive correlation among these three miRNAs, suggesting a coordinated regulatory role. As Supplementary Fig. S2 shows, the most significant pathways emerging from this analysis in terms of FDR included those involved in 'Regulation of RUNX1 Expression and Activity’, 'Transcriptional activity of SMAD2/SMAD3/SMAD4 Heterotrimer’, and 'Signaling by TGF-beta Receptor Complex’. Other notable terms indicated a shared role in ‘SUMOylation’, 'post-translational silencing by small RNAs’, and fundamental aspects of 'Gene expression (Transcription)'. These findings highlight specific mechanistic hubs where the regulatory actions of these miRNAs converge.

The Pathway Union analysis, visualized as a heatmap (Supplementary Fig. S3), revealed each miRNA’s individual contribution to the significantly enriched Reactome pathways. This approach showed varying enrichment intensities (−log10(FDR) values) among the miRNAs for specific terms, highlighting both unique and shared regulatory patterns.

## Discussion

This study assessed the potential of circulating plasma miR-21-5p, miR-23a-3p, and miR-26a-5p, as biomarkers in an older population, aiming to identify associations with clinical, functional, and laboratory measures to enhance our understanding of their relevance to age-related health status. As outlined in the introduction, these miRNAs were selected based on strong evidence linking them to key aging hallmarks, including mitochondrial dysfunction, genomic instability, inflammation, and metabolic dysregulation (see references in the Introduction section).

Consistent with previous findings ^24,43^ an age-related increase in the expression of miR-21-5p, miR-23a-3p was observed. Notably, an age-associated increase in miR-26a-5p levels was also detected, a novel finding not previously reported to our knowledge. We further examined the distribution of participants across age tertiles and found a relatively balanced representation across the three groups, suggesting that the observed age-related changes are linear and not driven by any specific subset of participants.

All three miRNAs exhibited significant associations with multiple aging-related endophenotypes and showed strong intercorrelations, suggesting their involvement in shared or converging biological pathways.

Our results indicated that both miR-21-5p and miR-23a-3p levels were significantly elevated in individuals classified as frail. Notably, only miR-23a-3p showed a significant inverse correlation with hand grip strength, a well-established indicator of physical performance in older adults. This finding suggests that while both miRNAs are associated with frailty as a multidimensional entity, miR-23a-3p might be more specifically linked to musculoskeletal decline. These observations are consistent with prior studies reporting elevated levels of miR-21-5p and miR-23a-3p in frail individuals ^44,45^, as well as in bone tissue from osteoporotic patients ^46^ and in patients with osteoarthritis ^47,48^, a common cause of functional deterioration in older adults which shows a correlation and causal relationship with frailty ^49,50^.

Mir-21-5p exhibited a strong and consistent association with markers of impaired renal function, including elevated serum creatinine and BUN, along with reduced eGFR. This relationship was particularly evident in participants with moderate to severe renal impairment (eGFR ≤ 45 ml/min), suggesting its potential utility as a biomarker of renal disease severity. These results align with prior evidence implicating miR-21-5p in kidney injury and fibrosis, reinforcing its role as an indicator of renal decline in older adults ^51–53^. Conversely, miR-23a-3p demonstrated no significant correlation with eGFR but was positively correlated with serum potassium levels, potentially reflecting disturbances in renal tubular function or systemic electrolyte imbalance, common features of renal dysfunction contributing to frailty ^54,55^. Interestingly, elevated miR-23a-3p expression has also been reported in renal fibrotic tissues ^56^.

The co-occurrence of elevated miR-21-5p and miR-23a-3p levels in individuals with both frailty and renal dysfunction is particularly noteworthy. Frailty, characterized by reduced physiological reserve and increased vulnerability to adverse health outcomes ^32^, has gained increasing relevance in nephrology, with multiple studies highlighting its association with declining renal function ^57–59^. In line with these reports, frail individuals in our cohort exhibited significantly lower eGFR values than their non-frail counterparts. However, in a post-hoc multivariable analysis adjusting for eGFR, the association between miR-21-5p and frailty remained significant, indicating that this relationship is not solely mediated by renal impairment (data not shown). This suggests that miR-21-5p may reflect broader biological mechanisms relevant to both frailty and renal decline.

Regarding miR-26a-5p, although its association with frailty did not reach statistical significance (*p* = 0.11), it was significantly correlated with clinical and functional indicators of vulnerability. Higher miR-26a-5p levels were associated with lower ADL scores and higher CIRS scores. Recent studies have identified miR-26a-5p as upregulated in various pathological conditions associated with systemic inflammation, tissue degeneration, and chronic disease. These include disorders like Alzheimer’s disease and coronary artery disease ^60,61^, inflammatory conditions such as rheumatoid arthritis and asthma ^22,62^, and degenerative processes observed in disc injury models ^63^. The consistent upregulation of miR-26a-5p across diverse diseases and tissues suggests a possible role in mediating or reflecting multisystem dysfunction, supporting its association with pathophysiological states reflected in clinical indices of functional status and multimorbidity, such as ADL and CIRS. From a biochemical standpoint, miR-26a-5p showed a pattern consistent with systemic dysregulation. It was positively correlated with blood urea nitrogen (BUN) and serum potassium, suggesting a role in non-glomerular aspects of renal impairment. Furthermore, it showed a negative association with total protein, hemoglobin, and hematocrit levels. These findings, which parallel those for miR-23a-3p and miR-21-5p (the latter also positively correlated with lymphocyte percentage), are consistent with anemia and a pro-inflammatory state, which are clinical signs of systemic dysregulation.

Overall, in our cohort of older adults, miR-21-5p, miR-23a-3p, and miR-26a-5p were all positively correlated with functional decline phenotypes. Stratified analyses by sex, age, and comorbidity level further supported the robustness of the observed associations. The direction and strength of the correlations were largely consistent across subgroups, suggesting that the identified miRNA patterns are not confined to specific demographic or clinical strata.

Interestingly, these three miRNAs were also significantly correlated with each other, suggesting the involvement of shared molecular pathways. To investigate this hypothesis, we conducted an in-silico pathway analysis using DIANA-miRPath v.4. The Reactome-based enrichment identified a significant overlap in the biological pathways targeted by these miRNAs, notably including the TGF-β, SMAD, and RUNX1 signaling cascades.

Among these, TGF-β signalling emerged as a central node. This cytokine regulates key biological processes such as cellular senescence, tissue repair, immune modulation, and fibrogenesis, all of which contribute to chronic organ dysfunction and age-related decline ^64–66^. Its canonical activation via SMAD proteins promotes extracellular matrix deposition and tissue stiffening, hallmark features of fibrosis in renal, hepatic, and pulmonary systems ^67,68^. The dysregulation of TGF-β/SMAD signaling has also been associated with muscle atrophy and chronic inflammation, key features of frailty ^65,69,70^.

Multiple studies have demonstrated that miR-21-5p functions as a critical mediator of TGF-β–induced renal fibrosis, both by targeting Smad7, a negative regulator of the pathway, and by modulating PTEN/AKT and ERK/MAPK cascades ^71,72^. By targeting the TGF-beta/SMAD7-SMAD2/3 signalling pathway, miR-21 also regulates skeletal muscle atrophy and fibrosis ^73^. Its association with renal parameters and frailty in our study further supports its role as a mediator of impaired tissue function and organ failure in aging.

Similarly, miR-23a-3p has been shown to downregulate SnoN, a suppressor of TGF-β signaling, thereby facilitating pro-fibrotic mechanisms such as epithelial–mesenchymal transition (EMT) and matrix accumulation ^56^. Conversely, its inhibition can be protective, as seen in models of osteoarthritis, where it modulates the TGF-β/Smad–Grem1 axis ^74^.

Although the connection between miR-26a-5p and TGF-β is less direct, emerging evidence points to its role in fibrotic signaling networks. In hepatic fibrosis, inhibition of miR-26a-5p enhances hepatocyte growth factor (HGF) production, an antagonist of TGF-β–induced fibrosis ^75^. Müller-Deile et al. ^76^ demonstrated that overexpression of miR-26a-5p contributes to impairment of the filtration barrier function impacting on glomerular VEGF expression via suppression of PIK3C2α, known to converge functionally with TGF-β ^77^.

Among the enriched pathways, RUNX1 (RUNX Family Transcription Factor 1) signaling emerged with the highest enrichment score, although its functional connection to all three miRNAs is less established. Nonetheless, as a transcription factor involved in several processes, such as haematopoietic cell development and function and inflammation ^78,79^, its appearance suggests additional layers of transcriptional regulation potentially shared by these miRNAs. RUNX1 is known to interface with TGF-β and SMAD pathways in several regulatory networks ^80–82^, suggesting potential functional interplay worth further exploration.

### Limitations

This study has several limitations that should be considered when interpreting the results. First, the relatively small sample size and predominantly female composition may limit the generalizability of our findings, although the demographic structure accurately reflects the population recruited. Furthermore, recruitment from nursing homes, while ensuring standardized living conditions, may have introduced a selection bias towards individuals with a higher comorbidity burden and frailty status. Second, the study employed a cross-sectional design. This inherent limitation, coupled with the reliance on correlation and multiple regression analyses, precludes the inference of causality or the exploration of dynamic relationships between the circulating miRNAs and aging parameters. While we performed exploratory bioinformatic analyses to generate mechanistic hypotheses (e.g., TGF-β/SMAD and RUNX1 pathways), we acknowledge the lack of experimental validation in this report. Third, while the observed correlations were statistically significant, their moderate effect size indicates that factors beyond age and sex likely contribute to the associations. Although we extensively analysed a wide range of clinical and functional parameters and performed subgroup and stratification analyses (by sex, age, and comorbidity), residual confounding from unmeasured variables (such as specific nutritional components or detailed inflammatory pathways) cannot be excluded. Finally, while the subgroup analyses supported the robustness and consistency of the findings, the reduced statistical power within these smaller strata limits our ability to draw definitive conclusions for specific subgroups.

Despite these limitations, our study provides a valuable, data-driven, hypothesis-generating framework and identifies specific candidate biomarkers that warrant further investigation in future larger, longitudinal, and mechanistic studies.

## Conclusions

Our findings demonstrate that circulating miR-21-5p, miR-23a-3p, and miR-26a-5p are associated with functional decline and renal impairment in older adults. The consistency of these associations was further supported by multivariable analyses and stratification across different sex, age, and comorbidity subgroups. In silico analyses revealed a shared enrichment in biologically plausible pro-fibrotic and inflammatory pathways, specifically highlighting the involvement of TGF-β/SMAD and RUNX1 signaling. This strongly supports the hypothesis that these miRNAs act as upstream modulators of inflammation-mediated fibrogenic processes.

We propose that fibrosis may represent a unifying biological mechanism that links these three microRNAs to systemic functional decline and renal damage in older adults. While the mechanistic links require experimental validation, the combination of our clinical correlations and pathway insights provides a strong, data-driven framework. These miRNAs represent valuable hypothesis-generating biomarkers reflecting complex molecular processes underlying aging and its related comorbidities. Future studies should prioritize the experimental clarification of their roles and explore their prognostic or therapeutic value in geriatric populations.

## Supplementary Information

Below is the link to the electronic supplementary material.

Supplementary Material 1

Supplementary Material 2

Supplementary Material 3

Supplementary Material 4

Supplementary Material 5

Supplementary Material 6

Supplementary Material 7

## Data Availability

The datasets generated through this work are available upon reasonable request from the corresponding author.
