# Supplementary figures and images for: Circulating microRNAs miR-21-5p, miR-23a-3p and miR-26a-5p reflect clinical and molecular features of aging
Source: Sci Rep. 2025 Dec 17;16:2690. doi: 10.1038/s41598-025-32412-0 (PMC12823579; doi:10.1038/s41598-025-32412-0)

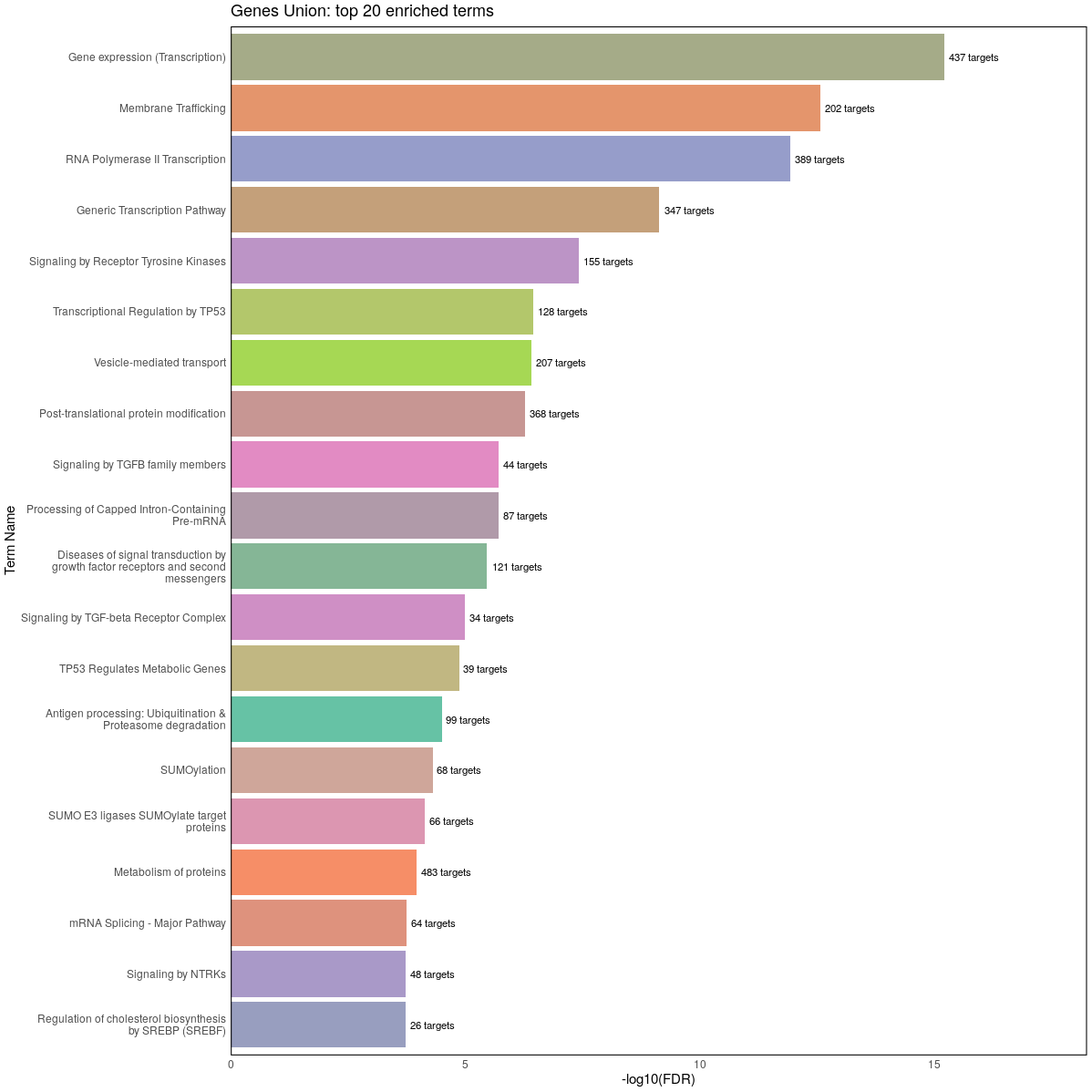

Supplement: Supplementary file 1 — Supplementary Material 1 [file 41598_2025_32412_MOESM1_ESM.tiff]

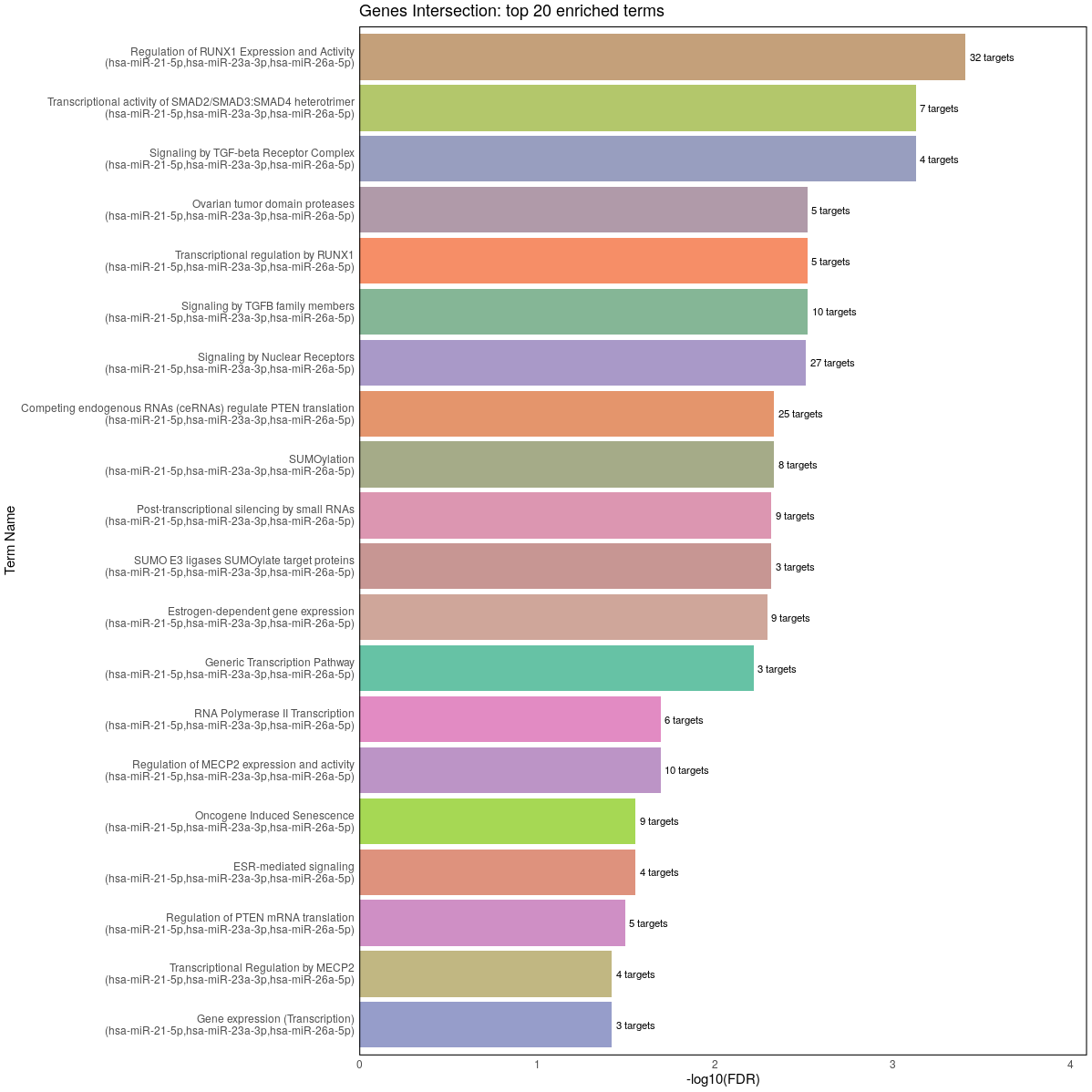

Supplement: Supplementary file 2 — Supplementary Material 2 [file 41598_2025_32412_MOESM2_ESM.tiff]

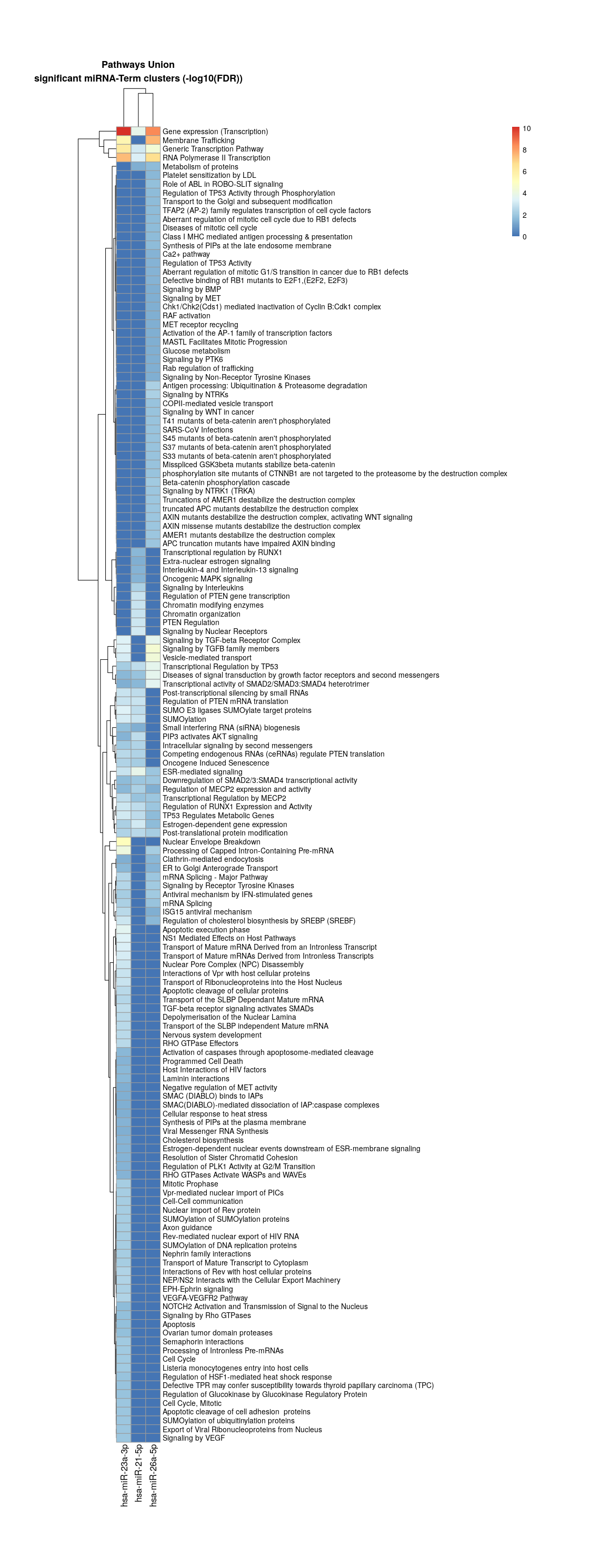

Supplement: Supplementary file 3 — Supplementary Material 3 [file 41598_2025_32412_MOESM3_ESM.tiff]
