# Supplementary material for: Circulating microRNAs miR-21-5p, miR-23a-3p and miR-26a-5p reflect clinical and molecular features of aging
Source: Sci Rep. 2025 Dec 17;16:2690. doi: 10.1038/s41598-025-32412-0 (PMC12823579; doi:10.1038/s41598-025-32412-0)
Supplement: Supplementary file 4 — Supplementary Material 4 [file 41598_2025_32412_MOESM4_ESM.docx]

Figure S1. Top 20 enriched terms from Gene Union analysis (Reactome database)

The 20 most significantly enriched Reactome terms from the 'Gene Union' analysis. The bar chart shows the top 20 enriched Reactome pathways/terms, identified by considering all predicted target genes of at least one of the miRNAs (hsa-miR-21-5p, hsa-miR-23a-3p, hsa-miR-26a-5p) using DIANA-miRPath v4.0. The Y-axis indicates the name of the pathway/term. The X-axis represents statistical significance as -log10(FDR) (False Discovery Rate corrected); higher values indicate greater significance. The number above each bar indicates the number of union target genes contributing to the enrichment of that pathway.

Figure S2. Top 20 enriched terms from Gene Intersection analysis (Reactome database)

The 20 most significantly enriched Reactome terms from the 'Gene Intersection' analysis. The bar chart shows the top 20 enriched Reactome pathways/terms, identified by considering only the genes that are predicted targets of all three miRNAs (hsa-miR-21-5p, hsa-miR-23a-3p, hsa-miR-26a-5p) simultaneously, using DIANA-miRPath v4.0. The Y-axis indicates the name of the pathway/term. The X-axis represents statistical significance as -log10(FDR); higher values indicate greater significance. The number above each bar indicates the number of common target genes contributing to the enrichment of that pathway.

Figure S3. Significant miRNA-Term clusters from Pathway Union analysis (Reactome database)

Heatmap from the 'Pathway Union' analysis showing Reactome term enrichment by individual miRNAs. The heatmap displays Reactome pathways/terms significantly enriched based on the meta-analysis of individual enrichments for hsa-miR-21-5p, hsa-miR-23a-3p, and hsa-miR-26a-5p, performed with DIANA-miRPath v4.0. Each row represents a pathway/term, and each column represents one of the miRNAs. The color of each cell indicates the significance of enrichment of that pathway by the specific miRNA, measured as -log10(FDR), according to the displayed color scale. Warmer colors (red) indicate higher significance. The dendrograms show the hierarchical clustering of pathways and miRNAs with similar enrichment profiles.
