# Supplementary material for: Circulating microRNAs miR-21-5p, miR-23a-3p and miR-26a-5p reflect clinical and molecular features of aging
Source: Sci Rep. 2025 Dec 17;16:2690. doi: 10.1038/s41598-025-32412-0 (PMC12823579; doi:10.1038/s41598-025-32412-0)
Supplement: Supplementary file 5 — Supplementary Material 5 [file 41598_2025_32412_MOESM5_ESM.docx]

**Supplementary Table S2.** ANCOVA results for circulating miRNAs stratified by age groups (Below vs Above Median Age), with adjustment for sex as a covariate. The analysis evaluates the effect of age group on miRNA expression in relation to multiple clinical and functional parameters.

| miRNA | Clinical Parameter | F (df1, df2) | p-value | ηp² (Effect Size) |
| --- | --- | --- | --- | --- |
| miR-21-5p | BUN^a^ | 5.47 (1,188) | 0.020 | 0.028 |
| miR-21-5p | sCr^a^ | 4.49 (1,201) | 0.035 | 0.022 |
| miR-21-5p | eGFR | 5.85 (1,201) | 0.016 | 0.028 |
| miR-21-5p | TP^a^ | 7.37 (1,182) | 0.007 | 0.039 |
| miR-21-5p | LYM | 6.69 (1,197) | 0.010 | 0.033 |
| miR-21-5p | RBC | 6.23 (1,200) | 0.012 | 0.030 |
| miR-21-5p | HCT^a^ | 6.62 (1,199) | 0.011 | 0.011 |
| miR-21-5p | Hb^a^ | 5.47 (1,199) | 0.020 | 0.027 |
| miR-21-5p | Frailty | 5.11 (1,204) | 0.025 | 0.024 |
|  |  |  |  |  |
| miR-23a-3p | TP^a^ | 9.40 (1,181) | 0.003 | 0.049 |
| miR-23a-3p | K | 5.67 81,196) | 0.018 | 0.028 |
| miR-23a-3p | RBC | 5.79 (1,199) | 0.017 | 0.028 |
| miR-23a-3p | HCT^a^ | 7.15 (1,198) | 0.008 | 0.035 |
| miR-23a-3p | Hb^a^ | 6.02 (1,198) | 0.015 | 0.030 |
| miR-23a-3p | Frailty | 8.46 (1,203) | 0.004 | 0.040 |
| miR-23a-3p | HGS^a^ | 9.22 (1,153) | 0.003 | 0.057 |
|  |  |  |  |  |
| miR-26a-5p | BUN^a^ | 8.88 (1,187) | 0.003 | 0.045 |
| miR-26a-5p | TP^a^ | 14.34 (1,181) | <0.001 | 0.073 |
| miR-26a-5p | K | 7.59 (1,196) | 0.006 | 0.037 |
| miR-26a-5p | RBC | 4.46 (1,199) | 0.036 | 0.022 |
| miR-26a-5p | HCT^a^ | 9.15 (1,198) | 0.003 | 0.044 |
| miR-26a-5p | Hb^a^ | 9.33 (1,198) | 0.003 | 0.045 |
| miR-26a-5p | ADL | 6.19 (1,202) | 0.014 | 0.030 |
| miR-26a-5p | CIRS^a^ | 6.65 81,195) | 0.011 | 0.033 |

(a) log-transformed parameters. The F and p-values correspond to the main effect of the Clinical Parameter. The F-test for the interaction term (Clinical Parameter × age) was non-significant for all models (pInt​≥0.05).

Post-hoc comparisons were not applicable since the stratification included two levels. Partial eta squared (ηp²) indicates small-to-moderate effect sizes (range 0.011–0.077).

*Abbreviations*: BUN = Blood Urea Nitrogen, sCr = Serum Creatinine, eGFR = Estimated Glomerular Filtration Rate, TP = Total Protein, K = Potassium, LYM = Lymphocytes, RBC = Red Blood Cells, HCT = Hematocrit, Hb = Hemoglobin, HGS = Hand Grip Strength, ADL = Activities of Daily Living, CIRS = Cumulative Illness Rating Scale.
