# Supplementary material for: Circulating microRNAs miR-21-5p, miR-23a-3p and miR-26a-5p reflect clinical and molecular features of aging
Source: Sci Rep. 2025 Dec 17;16:2690. doi: 10.1038/s41598-025-32412-0 (PMC12823579; doi:10.1038/s41598-025-32412-0)
Supplement: Supplementary file 6 — Supplementary Material 6 [file 41598_2025_32412_MOESM6_ESM.docx]

**Supplementary Table S1.** ANCOVA results for circulating miRNAs stratified by sex (male/female), with adjustment for age as a covariate. The analysis evaluates the effect of sex on miRNA expression in relation to multiple clinical parameters.

| miRNA | Clinical Parameter | F (df1, df2) | p-value | ηp² (Effect Size) |
| --- | --- | --- | --- | --- |
| miR-21-5p | BUN^a^ | 4.97 (1,188) | 0.027 | 0.026 |
| miR-21-5p | sCr^a^ | 4.45 (1,201) | 0.036 | 0.022 |
| miR-21-5p | eGFR | 5.38 (1,201) | 0.021 | 0.026 |
| miR-21-5p | TP^a^ | 5.89 (1,182) | 0.016 | 0.031 |
| miR-21-5p | LYM | 7.40 (1,197) | 0.007 | 0.036 |
| miR-21-5p | RBC | 5.24 (1,200) | 0.023 | 0.026 |
| miR-21-5p | HCT^a^ | 5.83 (1,199) | 0.017 | 0.028 |
| miR-21-5p | Hb^a^ | 4.74 (1,199) | 0.031 | 0.023 |
| miR-21-5p | Frailty | 4.51 (1,204) | 0.035 | 0.022 |
|  |  |  |  |  |
| miR-23a-3p | TP^a^ | 6.36 (1,181) | 0.013 | 0.034 |
| miR-23a-3p | K | 5.33 (1,196) | 0.022 | 0.026 |
| miR-23a-3p | RBC | 3.95 (1,203) | 0.048 | 0.019 |
| miR-23a-3p | HCT^a^ | 5.35 (1,198) | 0.022 | 0.026 |
| miR-23a-3p | Hb^a^ | 4.39 (1,198) | 0.037 | 0.022 |
| miR-23a-3p | Frailty | 6.14 (1,203) | 0.014 | 0.029 |
| miR-23a-3p | HGS^a^ | 8.03 (1,153) | 0.005 | 0.050 |
|  |  |  |  |  |
| miR-26a-5p | BUN^a^ | 6.92 (1,187) | 0.009 | 0.036 |
| miR-26a-5p | TP^a^ | 10.26 (1,181) | 0.002 | 0.054 |
| miR-26a-5p | K | 7.19 (1,196) | 0.008 | 0.035 |
| miR-26a-5p | RBC | 2.65 (1,199) | 0.090 | 0.013 |
| miR-26a-5p | HCT^a^ | 6.94 (1,198) | 0.009 | 0.034 |
| miR-26a-5p | Hb^a^ | 7.13 (1,198) | 0.008 | 0.035 |
| miR-26a-5p | ADL | 4.63 (1,202) | 0.033 | 0.022 |
| miR-26a-5p | CIRS^a^ | 6.47 (1,195) | 0.012 | 0.032 |

(a) log-transformed parameters.

The F and p-values correspond to the main effect of the Clinical Parameter. The F-test for the interaction term (Clinical Parameter × Sex) was non-significant for all models (pInt​≥0.05), except for ADL, where a significant interaction between ADL and sex was observed for miR-26a-5p (pInt​<0.05), suggesting a possible sex-specific effect.

Post-hoc comparisons were not applicable since the stratification included only two levels. Partial eta squared (ηp²) indicates small-to-moderate effect sizes (range = 0.013–0.055).

*Abbreviations*: BUN = Blood Urea Nitrogen, sCr = Serum Creatinine, eGFR = Estimated Glomerular Filtration Rate, TP = Total Protein, K = Potassium, LYM = Lymphocytes, RBC = Red Blood Cells, HCT = Hematocrit
