# Supplementary material for: Circulating microRNAs miR-21-5p, miR-23a-3p and miR-26a-5p reflect clinical and molecular features of aging
Source: Sci Rep. 2025 Dec 17;16:2690. doi: 10.1038/s41598-025-32412-0 (PMC12823579; doi:10.1038/s41598-025-32412-0)
Supplement: Supplementary file 7 — Supplementary Material 7 [file 41598_2025_32412_MOESM7_ESM.docx]

**Supplementary Table S3.** ANCOVA results for circulating miRNAs stratified by comorbidity level (Low vs High CIRS Score), with adjustment for sex and age as covariates. The analysis evaluates the effect of comorbidity burden on miRNA expression in relation to multiple clinical and functional parameters.

| miRNA | Clinical Parameter | F (df1, df2) | p-value | ηp² (Effect Size) |
| --- | --- | --- | --- | --- |
| miR-21-5p | BUN^a^ | 3.89 (1,179) | 0.050 | 0.021 |
| miR-21-5p | sCr^a^ | 4.18 (1,192) | 0.042 | 0.021 |
| miR-21-5p | eGFR | 5.72 (1,192) | 0.018 | 0.029 |
| miR-21-5p | TP^a^ | 5.35 (1,173) | 0.022 | 0.030 |
| miR-21-5p | LYM | 8.17 (1,189) | 0.005 | 0.041 |
| miR-21-5p | RBC | 4.52 (1,192) | 0.035 | 0.023 |
| miR-21-5p | HCT^a^ | 4.68 (1,191) | 0.032 | 0.024 |
| miR-21-5p | Hb^a^ | 3.70 (1,191) | 0.056 | 0.056 |
| miR-21-5p | Frailty | 3.06 (1,195) | 0.082 | 0.015 |
|  |  |  |  |  |
| miR-23a-3p | TP^a^ | 5.81 (1,172) | 0.017 | 0.033 |
| miR-23a-3p | K | 5.78 (1,187) | 0.017 | 0.030 |
| miR-23a-3p | RBC | 3.18 (1,191) | 0.076 | 0.016 |
| miR-23a-3p | HCT^a^ | 3.98 (1,190) | 0.047 | 0.021 |
| miR-23a-3p | Hb^a^ | 3.31 (1,190) | 0.075 | 0.017 |
| miR-23a-3p | Frailty | 4.39 (1,194) | 0.038 | 0.022 |
| miR-23a-3p | HGS^a^ | 8.47 (1,147) | 0.004 | 0.054 |
|  |  |  |  |  |
| miR-26a-5p | BUN^a^ | 5.39 (1,179) | 0.021 | 0.029 |
| miR-26a-5p | TP^a^ | 7.08 (1,172) | 0.009 | 0.040 |
| miR-26a-5p | K | 7.83 (1,187) | 0.006 | 0.040 |
| miR-26a-5p | RBC | 1.21 (1,191) | 0.273 | 0.006 |
| miR-26a-5p | HCT^a^ | 4.02 (1,190) | 0.046 | 0.021 |
| miR-26a-5p | Hb^a^ | 4.26 81,190) | 0.040 | 0.022 |
| miR-26a-5p | ADL | 2.12 (1,193) | 0.147 | 0.011 |

(a) log-transformed parameters. The F and p-values correspond to the main effect of the Clinical Parameter. The F-test for the interaction term (Clinical Parameter × comorbidity) was non-significant for all models (pInt​≥0.05). Post-hoc comparisons were not applicable since the stratification included two levels. Partial eta squared (ηp²) indicates small-to-moderate effect sizes (range 0.011–0.054). *Abbreviations*: BUN = Blood Urea Nitrogen, sCr = Serum Creatinine, eGFR = Estimated Glomerular Filtration Rate, TP = Total Protein, K = Potassium, LYM = Lymphocytes, RBC = Red Blood Cells, HCT = Hematocrit, Hb = Hemoglobin, HGS = Hand Grip Strength, ADL = Activities of Daily Living, CIRS = Cumulative Illness Rating Scale.
